# Goats are more susceptible to *Haemonchus contortus* infection than sheep under similar experimental settings

**DOI:** 10.1038/s41598-024-74112-1

**Published:** 2024-10-25

**Authors:** Desta Risa, Gezahegne Mamo, Hika Waktole, Geremew Haile, Getachew Terefe

**Affiliations:** 1 School of Veterinary Medicine, Wollega University, Nekemte, Ethiopia; 2 Department of Microbiology, Immunology and Veterinary Public Health, College of Veterinary Medicine and Agriculture, Addis Ababa University, Bishoftu, Ethiopia; 3 Department of Pathology and Parasitology, College of Veterinary Medicine and Agriculture, Addis Ababa University, P. O. Box. 34, Bishoftu, Ethiopia

**Keywords:** Experimental infection, Goats, *H. Contortus*, *S*heep, Susceptibility, Biological techniques, Zoology

## Abstract

Due to differences in their feeding behavior, sheep and goats are often assumed to respond differently to helminth infections. The present study compared *Haemonchus contortus* infection profile between sheep and goats under the same experimental setting. Experimental infection was conducted using a randomized block design in four groups of intact sheep (InfSH and ConSH) and goats (InfG, and ConG). Groups InfSH and InfG (*N* = 7 each) received 10,000L3 of *H. contortus* whereas the control groups ConSH and ConG (*N* = 7 each) remained uninfected. Faecal egg counts and PCV were measured from Day 0 to day 56 post infection (PI). On day 56 PI, animals were humanely slaughtered and abomasal contents were recovered to measure worm burden, worm length and *in utero* egg count. The findings show that: (1) *Haemonchus* infected animals showed an increase in FEC starting from day 21 PI, (2) progressive reduction in PCV was registered from day 7 PI and continued to the end of the experiment while this remained at pre-infection levels in control groups, (3) FEC was much higher (*P* < 0.001) and PCV was significantly lower (*P* < 0.05) in infected goats than in infected sheep, (4) at necropsy, total worm burden with worm establishment rates of 63% and 28.87% were registered respectively for infected goats and sheep with significant difference (*P* < 0.05), (5) Female worms were significantly longer (*P* < 0.05) in InfG (22.8(± 1.2) compared to InfSH (20.5 ± 0.67 mm) while (6) Mean worm fecundity was 974.8 ± 239.4 and 1162.5 ± 89.4 respectively for groups InfSH and InfG with no significant difference (*P* = 0.07), and (7) Parasite traits such as worm burden, FEC and female worm length were well correlated in sheep whereas few such patterns were observed in goats. In conclusion, under the same experimental infection, Arsi-Bale goats are more susceptible to *H. contortus* infection than Arsi-Bale sheep and hence deserve special attention when they are forced to live on grazing rather than browsing.

## Introduction

Sheep and goats are known to play major role in the rural economy in many parts of Ethiopia. However, efforts to maximize the benefits have often been challenged by constraints such as low genetic performance and various diseases of economic and public health importance. Helminthosis caused by gastrointestinal nematodes is one of the diseases impeding productivity of small ruminants worldwide; leading to poor yield and significant economic losses in both commercial and the smallholder farming system^1,2^. Among gastrointestinal helminths *Haemonchus contortus*is one of the most prevalent and economically important parasites of small ruminants in tropical, subtropical and temperate regions of the world. Losses because of haemonchosis are attributed to decreased production, costs of prophylaxis and treatment and death of infected animals^3–5^.

Several researches in other countries have documented conflicting findings on the relative importance of gastrointestinal parasitism in sheep and goats. Tesfaheywet^6^and Tesfaheywet and Murga^7^ in Ethiopia have shown that under natural infections, sheep are more affected by *H. contortus*than goats as judged from fecal egg examination and adult worm recovery. Similarly, Beriajaya and Copeman^8^ reported higher burden and establishment rate in Javanese thin tail sheep than in Kacang cross Etawah goats experimentally infected with *H. contortus* and *Trichostrongylus colubriformis.*On the contrary, Muluneh et al.^9^ in Ethiopia and Choubisa and Jaroli^10^ in India reported that the problem is more prevalent and intensity of infection was much higher in naturally infected goats than in sheep while other reports from India and Pakistan failed to observe any significant difference between the two^11,12^. Such variations may be attributed to differences in resistance/susceptibility of the hosts and/or environmental/nutritional factors that affect animal feeding behavior or survival of free living stages of gastrointestinal nematode parasites^11–13^. What is common for most of these reports is that *H. contortus* infection was the most prevalent helminth parasite in both sheep and goats. This study compared the infection profile of *H. contortus* between Arsi-Bae sheep and Arsi-Bale goats under experimental conditions with grass hay and wheat bran feed sources.

## Materials and methods

### Experimental animals and experimental setting

The experimental study was conducted in the College of Veterinary Medicine and Agriculture, (CVMA) located at Bishoftu town from September 2019 to June 2020. Bishoftu town is found at 47 km Southeast of Addis Ababa. Twenty eight healthy 12–18 months old intact males Arsi-Bale sheep and goats weighing 15–25 Kg and reared under the traditional grazing management system were purchased from Assela open market (East Arsi Zone, Oromia Regional State). Health conditions were assessed based on rectal temperatures, conjunctival mucous membrane examination, absence of nasal and lacrimal discharges, oral lesions and any visually detectable physical abnormalities. During the experimental period, all animals were placed in a fly-proof experimental animal facility where they were allowed to acclimatize for one month and their health conditions were monitored. All animals were provided with grass hay and water ad-libitum and supplemented with wheat bran until the end of the experiment. Before the commencement of the experiment, all animals were dewormed with Ivermectin (Hebeiyuanzheng Pharmaceutical Co.,Ltd./ China) at 0.5 ml/25Kg, Praziquantel 15 mg/kg (APF, Ethiopia) and Triclabendazole 250 mg/25kg (Fasinex 250, EAP, Ethiopia) to remove any helminth parasites from previous natural infections. Fifteen days post deworming, fresh fecal samples were collected from all animals and examined by using floatation, sedimentation and Bearmann techniques to make sure that all are free of helminth eggs or larvae as per the method described in MAFF^14^. The experimental study was approved by the Animal Research Ethics Review Committee of the College of Veterinary Medicine and Agriculture (Ref. No. VM/ERC/30/2020). The experiments were performed according to the ARRIVE guidelines (PLoS Bio 8(6), e1000412, 2010).

### Experimental design

A randomized block design was used to undertake the experimental study. At the end of adaptation period, all animals (14 male sheep and 14 male goats) were weighed and ear tagged. Following blocking for body weight, they were allocated into four treatment groups: infected sheep (InfSH), infected goats (InfG), non-infected control sheep (ConSH) and non-infected control goats (ConG). Each group had seven animals housed in separate clean pens.

### Experimental parasites and animal infection

Adult female *Haemonchus contortus *worms were collected from the abomasa of naturally infected sheep and goats generously provided by Hashim Export Abattoir, Bishoftu. The parasites were recovered by passing the abomasal contents through a sieve of 150 μm diameter and were picked with wire loop. These parasites were crashed with a mortar and pistil to liberate the eggs. The homogenate was then mixed with helminth egg free cattle feces and incubated at room temperature ranging between 25 and 27 °C for 14 days with regular aeration and moisturization^15^. The infective larvae (L3) were harvested using modified Baermann technique as described by Kumsa^16^. The larvae were stored at 4 °C in tap water until used for the intended experimental infection of sheep and goats. Viability of larvae was confirmed under stereomicroscope before donor animals (one sheep and one goat) were infected for mass production of L3. Once the establishment of the infection was confirmed by fecal egg examination, large volumes of fecal materials were collected from the rectum for culturing as described above. About 200,000 L3 were then made ready for the experimental infection by mixing larvae harvested from both donor animals. Each animal in the two infection groups (InfSH and InfG) received 10,000 L3 in 15 ml of water on day zero (D0) while animals in control groups (ConSH and ConG) were drenched with equal volume of water^17^.

### Data collection

#### Fecal egg count (FEC)

Faecal samples were collected from the rectum of all experimental animals twice a week starting from D0 (day of infection) until the end of the experiment (D56) for detection and counting of fecal eggs according to the methods described by Taylor et al.^18^ and Zajac and Conboy^19^. Briefly, 3 g of the faecal sample was homogenised in 42 ml of salt solution (500 g NaCl in 1000 ml H_2_O: Specific gravity = 1.20) which makes a final volume of approximately 45 ml (1gram/15 ml). Eggs were counted by the McMaster technique. The eggs in the two chambers of the McMaster slide were counted, total values multiplied by a correction factor of 50 and the numbers of egg per gram of faeces (EPG) were determined according to the formula given by MAFF^14^.

#### Packed cell volumes (PCV)

Blood samples from each experimental group (*n* = 7/group) were collected weekly via jugular venipuncture in 4 ml EDTA coated vacutainer tubes (K3 EDTA 7.2 mg, Italy) from D0 until the end of the experiment (D56). Capillary tubes were filled with the blood samples in duplicates and centrifuged at 12,000 rpm for 3–5 min. Hematocrit capillary reader was used to register the %PCV.

#### Worm recovery

At the end of the experiment (D56), all sheep and goats (infected and control) were slaughtered humanely by intravenous injection of 10 mg/kg barbiturate. The abomasa were removed and tied at both ends, opened along the greater curvature and the contents were poured onto a tray. This was thoroughly washed by passing through a sieve of 150 μm apertures and the whole matter remaining on the sieve was collected in a bottle containing 70% alcohol the volume of which was later adjusted to 500 ml. The mucosal surface was carefully washed and also socked in saline solution for 6 h and all the washings were collected, preserved in 70% alcohol and adjusted similarly to 500 ml to recover immature worms embedded in the mucosa^17^.

#### Worm count, measurement and female fecundity

Worms were counted in a 10% aliquot of the preserved materials and the counts were multiplied by 10 to obtain worm burden/animal. Worms were classified as adult male and female, immature male and female including L4 stages. The lengths of arbitrarily selected 30 adult female and 30 adult male worms were measured to the nearest millimetre using a ruler for each infected animal. By slightly modifying the method described by Kloosterman et al.^20^, measured female worms were individually digested by socking in 200 µl mild bleaching agent (1.25% sodium hypochlorite) locally known as ‘*wuha agar*’ which is used for purifying drinking water. This solution was pretested and found that it had no effect on the eggs for at least 10 min. All eggs released from the uterus were then counted under a stereomicroscope with x4 magnification.

### Data management and analysis

Microsoft Excel 2010 database system was used for entry, coding and simple calculation of recorded data. Data was analyzed using StataCorp: Statistical Software for data science Statistical version 13 (https://www.stata.com/support/updates/stata13.html) and R-project for statistical computing version 3.5.1. The kinetics of egg excretion and packed cell volumes were compared between the four experimental groups using analysis of variance (ANOVA). The Pearson correlation test was used for assessing the correlation between parameters. Comparisons of the number *H. contortus* worm populations and worm length at necropsy and the number of eggs *in utero* per female were performed using analysis of variance (ANOVA) and two sample t- test with *P* < 0.05 level of significance.

## Results

### Faecal egg count

**Fig. 1:**
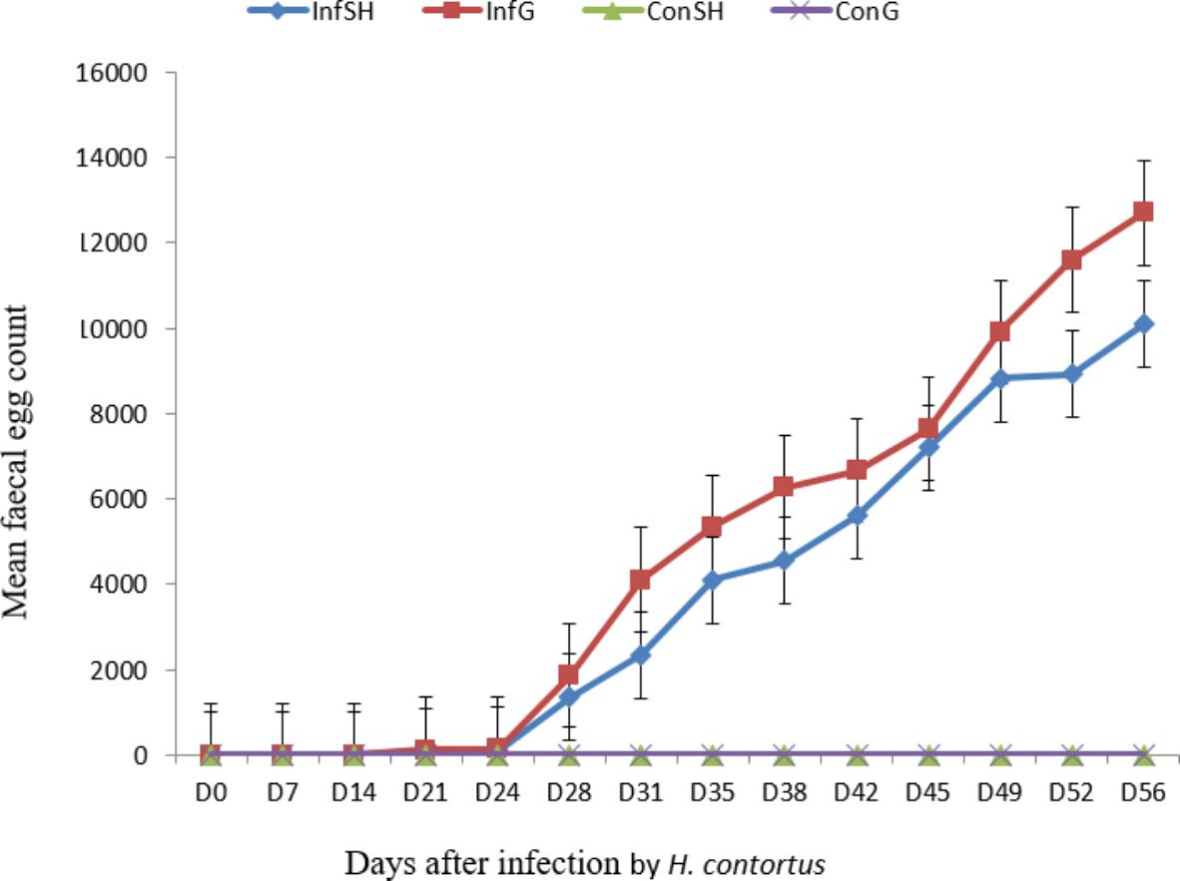
Mean FEC in sheep and goats experimentally infected with 10,000 *H. contortus* L3/animal (significant difference was observed on D56, *p* < 0.001).

No nematode egg was observed in fecal sample collected from the non-infected groups (ConSH and ConG) throughout the experimental period. The trend of egg excretion in *H. contortus* infected groups of sheep (InfSH) and goat (InfG) was similar starting on day 21 post infection (PI) and steadily increased up to the end of the experiment (Fig. 1). However, at the end of the experimental period (day 56), mean FEC in InfG (12701 ± 3228) was significantly higher (*p* < 0.001) compared to InfSH (10096 ± 3941). The cumulative mean number of fecal eggs in the seven weeks period was 4743 (± 4561.8) and 3801 (± 3775) for infected goats and sheep, respectively.

### Packed cell volumes (PCV)

**Fig. 2:**
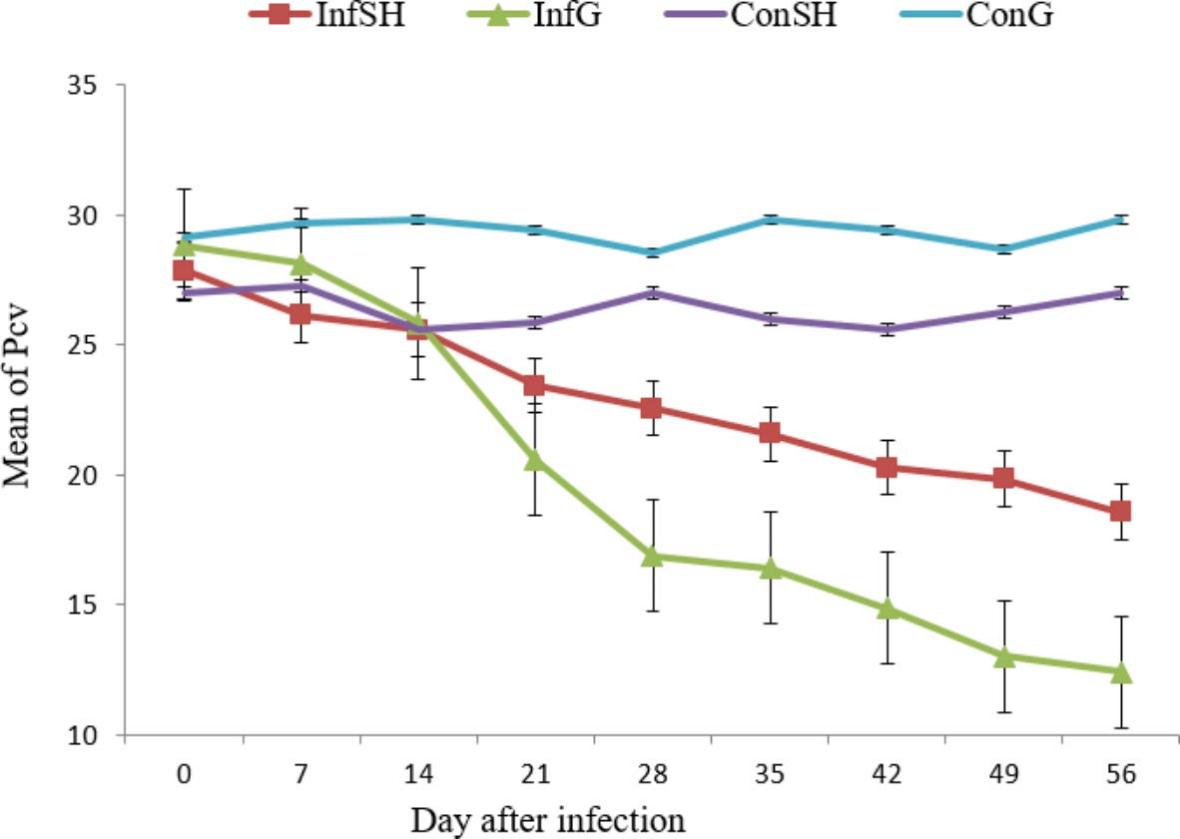
Mean packed cell volume (PCV) in the four experimental groups during the study period (Significant difference was observed starting from D21, *p* < 0.05).

The PCV values of uninfected control animals were similar to pre-infection levels throughout the trial period. On the other hand, a gradual decrease in PCV in infected groups started 7 days PI and continued declining until the end of the experiment with a mean PCV of 19.66% and 22.86% for infected goats and sheep respectively on day 56. The PCV of infected goats showed significant difference (*p* < 0.001) when compared with ConG all along the duration of the experiment (Fig. 2). Similarly, the PCV of infected sheep decreased and started to be significantly lower than that of ConSH at D21 (*p* < 0.001) PI. Comparison of infected sheep and goats revealed, significantly lower PCV in goats (*p* < 0.05) from day 21 PI onwards.

### Worm burden, worm length and female worm fecundity

No nematode worms were recovered from the abomasum of animals in the control groups. The mean number of *H. contortus* recovered from the abomasum of infected animals was higher in group InfG (6332 ± 2952) compared to those recovered from InfSH (2884 ± 2778) and the difference was significant (*p* = 0.043). The worms recovered from both infected groups were mainly adults with few immature (L4 and L5) stages. Worm establishment rates were 28.87% and 56% respectively for sheep and goats (Table 1). The mean number of egg *in utero* per female worm was 974.8 ± 239.4 and 1162.5 ± 89.4 for groups InfSH and InfG respectively. The count tend to be higher in goats than in sheep but, the difference was not significant (*p* = 0.07). The mean Male to female *H. contortus* sex ratios were 1:1.03 and I: 1.09 for sheep and goats respectively. The mean length in millimeters as calculated from measuring 30 male and 30 female worms/animal was 13.3 (± 0.59) for males and 20.5 (± 0.67)mm for females in sheep whereas it was 13.9 (± 0.76) for males and 22.8 (± 1.2) for females in goats. While there was no difference in the sizes of male parasites between the two hosts, female *H. contortus* collected from infected goats were significantly longer than those from sheep (*p* = 0.02).

**Table 1:** Total worm burden, sex composition, sex ratio, female length male length and number of eggs *in utero* of adult female *H.contortus*.

Parasite traits	Sheep (mean values ± SD)	Goats (mean values)	*P*-value
Male worm burden	1411.4 (± 1254)	3008.5	0.0184
Female worm burden	1457.14 (± 1503)	3154.3	0.0915
Worm sex ratio (M/F)	1:1.03	1:1.09	
Immature worms (L4 + L5)	15.7	27.14	0.1496
Fecundity (eggs/female)	974.8	1162 0.5	0.07
Female length (mm)	20.5	22.8	0.02
Male length (mm)	13.3	13.9	0.1174
Establishment rate (%)	28.87%	63.3%	0.0430

### Associations among different parasitological measurements

Pearson’s correlation coefficient (R) was calculated to see the association of various parasite traits as well as hematocrit values (Table 2). In sheep, mean FEC has shown significant positive correlation with total worm burden (*r* = 0.9533, *p* < 0.001) and number of female worms (*r* = 0.9324, *p* < 0.001). Similarly, female worm length increased with increasing worm burden (*r* = 0.7769, *p* < 0.05) whereas its association with mean FEC and *in utero* egg count was not significant. On the other hand, total worm count and number of female parasites tend to be negatively correlated with aggregated mean PCV (*p* = 0.07). In goats, such associations were significant only between female worm count and total worm burden (*r* = 0.8402, *p* < 0.05) and between total worm count and cumulative mean PCV (*r*= -0.7913, *p* < 0.05).

**Table 2:** Pearson’s correlation coefficient (R) among parasite traits and PCV in experimentally infected sheep and goats.

Parameters	PCV	Total burden	Female burden	Female length	EGG/female	FEC
*Sheep*						
Mean FEC	-0.5949	0.9533*	0.9324*	0.6277	0.2509	1
EGG/female	-0.6858	0.2398	0.2457	-0.1288	1	
female length	-0.3913	0.7769*	**0.7627**	1		
Female burden	**-0.7344**	0.9952*	1			
Total burden	**-0.7076**	1				
Mean PCV	1					
*Goat*						
Mean FEC	0.0946	-0.0186	0.4135	0.0452	-0.2269	1
EGG/female	0.6451	-0.5544	-0.3994	0.6494	1	
Female length	-0.0115	-0.1498	0.0684	1		
Female burden	-0.5887	0.8402*	1			
Total burden	-0.7913*	1				
Mean PCV	1					

* Significant correlation at *P* < 0.05.

Significant values are in bold.

## Discussion

### *H. contortus* worms developed much better in goats than in sheep

This study was conducted to compare infection profile of *H. contortus* and development of anemia arising from the infection between sheep and goats. In the bi-weekly fecal examination schedule, the first fecal egg was detected on D21 PI which is in agreement with the reports of other researchers in experimental infections with *H. contortus*^17,21^. Although both sheep and goats have excreted significant number of fecal eggs per gram of feces, the higher mean number of fecal egg count observed in infected goats compared to infected sheep suggests the parasites have established and/or developed better in the former than in the later. In this regards, the significantly higher values for worm burden, female worm length and *in utero* egg count of the parasite in goats may adequately explain the higher FEC in goats.

Research findings with regards to helminth parasite infections in goat and sheep are not conclusive enough to explain differences between the two hosts. Tesfaheywet^6^ and Tesfaheywet and Murga^7^ in Ethiopia have shown that sheep were more affected by gastrointestinal helminth parasite than goats as evaluated by fecal egg examination and adult worm recovery. On the contrary, Muluneh et al.^9^ in Ethiopia and Choubisa and Jaroli^10^ in India reported that the problem is more prevalent and intensity of infection was much higher in goats than in sheep while other reports from India and Pakistan did not observe any significant difference between the two host species^22,23^.

A review by Hoste et al.^24^ elaborated that although the brows feeding behavior of goats has the potential to help avoid contact with nematode infective larvae, if allowed to graze a contaminated pasture in the same way as sheep do, they can develop a more severe infection than sheep. The authors further explain that, such susceptibility of goats to gastrointestinal nematode infection could be ascribed to the inferior immune response they mount as a result of lower adaptation to the infection as compared to sheep. In our study, both sheep and goat were allowed to feed similar type of grass hay and wheat bran throughout the experimental period and both were equally exposed to *H. contortus* through experimental infection. Therefore, the higher development of the parasite (in terms of worm burden, female worm size and fecal egg count) in goats than in sheep can be explained by the failure of goats’ immune response to control the parasites. However, further study is needed if this situation also holds true among different breeds of goats and sheep.

A study in sheep breeds of Canary Islands have shown that acute inflammatory responses, complement activation, accelerated immune cell proliferation and subsequent tissue repair contributed to the development of host resistance to *H contortus*infection in the resistant Canaria hair breed of sheep compared to the susceptible Canaria breed^25^. The same report added that overexpression of both IL10 and IL13 mRNA molecules was more profound in the resistant breed than in the susceptible breed whereas the IL5 mRNA was upregulated by infection in the susceptible but barely detectable in the resistant suggesting that the differences have a genetic basis. Furthermore, the change in their nutrition from browse species to grass hay may have also caused some stress or denied the goats of self-medication advantage from selective browsing^24^.

Measurement of anemia as explained by PCV levels is an essential parameter which may be used to describe resistance against blood feeding nematode parasites^26^. Although both sheep and goats have developed anemia characteristic of *Haemonchus* infection, the decline in PCV value was more marked in infected goats. If the parasites have established and developed better in goats, it is inevitable that the mean PCV value in goats becomes much lower than those in sheep as a consequence of more blood feeding. The observed fall in PCV in animals infected with *H. contortus*was in agreement with the works of Terefe et al.^17^.

### Relationship of parasite traits varies between sheep and goats

Fecal egg count can be a function of worm number, female worm size and/or fecundity^17,27^. Such associations can be affected by host resistance or resilience^24,25^. In this study, fecal egg count was strongly linked to worm burden in sheep suggesting that egg count can be an indicator of the degree of parasite establishment. A similar finding was reported in different breeds of sheep naturally infected by various species of gastrointestinal helminth parasites including *H. contortus*^28^ and in cattle naturally infected by *H. placei* and and *Cooperia punctata*^29^. Moreover, the strong positive relationship between female worm length and worm burden suggests that suppression of parasite development by the host’s immune system affects both worm size and worm count simultaneously.

The degree of anemia as measured by PCV was negatively associated to worm count in sheep implying that if the host is unable to control *H. contortus*worm establishment, it will end up with tremendous reduction in PCV. Similar observations have been documented by previous reports in different breeds of sheep^30–32^. These findings are also in agreement with other studies on small ruminants^33^. Ona and Nawa^27^; Seat et al.^30^; Lacroux et al.^31^ and Terefe et al.^34^ further explained that such associations are affected by the hosts’ (species, breed, etc.) ability to mount cellular and humoral immune responses.

On the other hand, irrespective of the higher parasite establishment and development,, the correlation among parasite traits was very weak in goats; the only significant association observed being the negative relationship between worm burden and PCV. This strongly suggests that despite the similarity in their artificial infection, housing, feeding and overall management, Arsi-Bale goat and sheep differently reacted to *H. controtus*infection. This is complemented by the fact that significantly higher FEC, in utero egg count, female worm size and worm burden were registered in goats as compared to sheep. Ona and Nawa^27^ suggested that survival and development of nematode parasites in the host may vary depending on the species of parasite and animal model used for the study. In this regards, the finding in the current study disagrees with the observation of Cabare et al.^28^ who demonstrated significant positive relationship between fecal egg count and worm burden in goats. However, this study was conducted on goats naturally infected by several species of gastrointestinal helminthes with varying parasite fecundity and with no control over the number of infective larvae ingested.

## Conclusion

This study shown that goats were more affected than sheep on almost all parasitological and PCV parameters measured. In sheep, total worm burden was strongly associated with mean fecal egg count, female worm length and burden while in goats, the total worm burden affected only the degree of anemia as measured by PCV values, suggesting that goats and sheep reacted differently under similar conditions of the experimental infection. Hence, any helminth control program in small ruminants should give special attention to goats in situations where they are forced to graze pasture in the absence of brows species. Further study is needed to demonstrate the underlying host factors for the high susceptibility of goats compared to sheep.

## Data Availability

The raw data supporting the conclusions of this article will be made available by the authors, without undue reservation.
